# Chagas Disease, Migration and Community Settlement Patterns in Arequipa, Peru

**DOI:** 10.1371/journal.pntd.0000567

**Published:** 2009-12-15

**Authors:** Angela M. Bayer, Gabrielle C. Hunter, Robert H. Gilman, Juan G. Cornejo del Carpio, Cesar Naquira, Caryn Bern, Michael Z. Levy

**Affiliations:** 1 Division of Infectious Diseases, David Geffen School of Medicine, University of California at Los Angeles, Los Angeles, California, United States of America; 2 Asociación Benéfica Proyectos en Informática, Salud, Medicina y Agricultura (AB PRISMA), Lima, Peru; 3 Department of International Health, Johns Hopkins Bloomberg School of Public Health, Baltimore, Maryland, United States of America; 4 Dirección Regional del Ministerio de Salud, Arequipa, Peru; 5 Universidad Peruana Cayetano Heredia, Lima, Peru; 6 Division of Parasitic Diseases, Centers for Disease Control and Prevention, Atlanta, Georgia, United States of America; 7 Center for Clinical Epidemiology and Biostatistics, Department of Biostatistics and Epidemiology, University of Pennsylvania School of Medicine, Philadelphia, Pennsylvania, United States of America; 8 Fogarty International Center, National Institutes of Health, Bethesda, Maryland, United States of America; Ghana Health Service, Ghana

## Abstract

**Background:**

Chagas disease is one of the most important neglected tropical diseases in the Americas. Vectorborne transmission of Chagas disease has been historically rare in urban settings. However, in marginal communities near the city of Arequipa, Peru, urban transmission cycles have become established. We examined the history of migration and settlement patterns in these communities, and their connections to Chagas disease transmission.

**Methodology/Principal Findings:**

This was a qualitative study that employed focus group discussions and in-depth interviews. Five focus groups and 50 in-depth interviews were carried out with 94 community members from three shantytowns and two traditional towns near Arequipa, Peru. Focus groups utilized participatory methodologies to explore the community's mobility patterns and the historical and current presence of triatomine vectors. In-depth interviews based on event history calendars explored participants' migration patterns and experience with Chagas disease and vectors. Focus group data were analyzed using participatory analysis methodologies, and interview data were coded and analyzed using a grounded theory approach. Entomologic data were provided by an ongoing vector control campaign. We found that migrants to shantytowns in Arequipa were unlikely to have brought triatomines to the city upon arrival. Frequent seasonal moves, however, took shantytown residents to valleys surrounding Arequipa where vectors are prevalent. In addition, the pattern of settlement of shantytowns and the practice of raising domestic animals by residents creates a favorable environment for vector proliferation and dispersal. Finally, we uncovered a phenomenon of population loss and replacement by low-income migrants in one traditional town, which created the human settlement pattern of a new shantytown within this traditional community.

**Conclusions/Significance:**

The pattern of human migration is therefore an important underlying determinant of Chagas disease risk in and around Arequipa. Frequent seasonal migration by residents of peri-urban shantytowns provides a path of entry of vectors into these communities. Changing demographic dynamics of traditional towns are also leading to favorable conditions for Chagas disease transmission. Control programs must include surveillance for infestation in communities assumed to be free of vectors.

## Author Summary

Chagas disease affects 8–10 million people in the Americas. Although transmission was previously limited to the rural poor, Chagas increasingly affects urban populations, especially near the city of Arequipa, Peru. We interviewed residents of five communities to learn about why and when they migrated to the city and how their movements may link to Chagas vectors and to explore the settlement patterns of shantytowns and traditional towns. We found that migrants to shantytowns were unlikely to introduce Chagas vectors to the city upon first arrival. Frequent seasonal moves, however, took shantytown residents to valleys surrounding Arequipa where vectors are prevalent. In addition, the settlement pattern of shantytowns and the practice of raising domestic animals create a favorable environment for vectors. Finally, population loss and replacement by low-income migrants in one traditional town has created the human settlement pattern of a shantytown. This study exposes potential links between population dynamics and Chagas vector infestation. Suggested methods for improving vector control include focusing future vector surveillance in areas with mobile populations, creating educational campaigns for migrant workers to Chagas-endemic areas, and fomenting collaboration between the Arequipa Ministries of Health and Housing to ensure the inclusion of new shantytowns in vector surveillance.

## Introduction

Chagas disease, caused by infection with protozoan parasite *Trypanosoma cruzi*, causes more morbidity and mortality than any other parasitic disease in the Western Hemisphere [1]. *T. cruzi* is carried by numerous species of triatomine insects. Humans and other mammals usually become infected when the triatomine vector defecates during its blood meal, and fecal material containing the parasite is inoculated through the bite wound or mucous membranes [2]. Vector-borne transmission only occurs in the Americas, where 8–10 million people, including an estimated 192,000 Peruvians, are currently infected with *T. cruzi* [3],[4].

The member countries of the Southern Cone Initiative (INCOSUR) have worked since 1991 to eliminate household infestation with *Triatoma infestans*, the most important Chagas disease vector in the southern half of South America, through large-scale residual application of pyrethroid insecticides [5],[6]. Despite remarkable successes, major challenges remain to vector control, among them the increasing urbanization of the disease [7]. Chagas disease is traditionally associated with rural villages with adobe houses hospitable to *T. infestans* and other domestic vectors [8] and vector-borne transmission appears to be rare in urban settings [9]–[11]. However, in marginal communities of the city of Arequipa (pop. 750,000) in southern Peru, urban *T. cruzi* transmission cycles have become established [12],[13], and a vector control campaign has been in place in the city of Arequipa since 2002 [13].

The settlement and migration patterns in and around cities therefore may be important to understanding the dynamics that make certain communities more susceptible to Chagas disease vectors [14]. Latin America has experienced an overwhelming phenomenon of urbanization due in most part to in-migration, and Peru is no exception [15],[16]. Few studies have directly examined migration and settlement patterns, and their connections to Chagas disease transmission. Here we use qualitative methods to explore the migration and settlement patterns, and their links with vector infestation, in different communities around the city of Arequipa.

## Methods

### Ethics statement

The research protocol was approved by the ethical review committees of the Asociación Benéfica PRISMA and the Johns Hopkins Bloomberg School of Public Health. All participants provided written informed consent prior to data collection, including consent for audio-recording.

### Study setting

**Figure 1:**
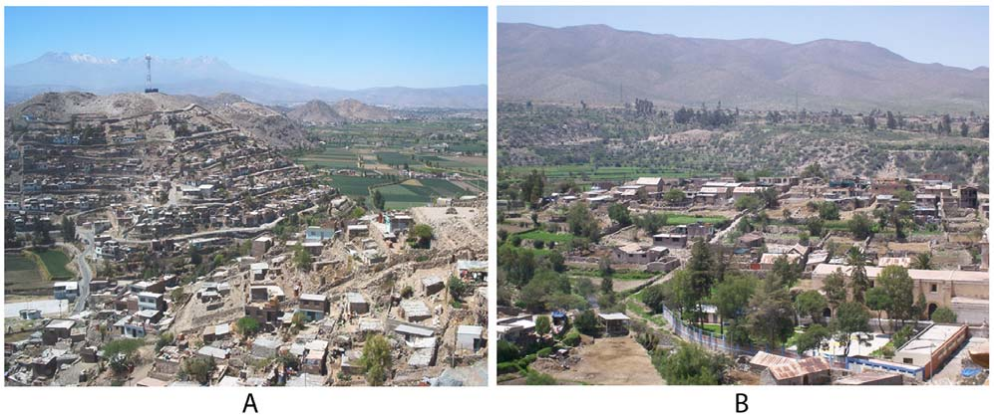
Photographs of the *pueblo joven* Guadalupe and *pueblo tradicional* Quequeña.

Arequipa is the second largest city in Peru, located in an arid zone 2,300 m above sea level [15]. The outskirts of the city contain hundreds of peri-urban *pueblos jóvenes* (young towns or shantytowns) and *pueblos tradicionales* (traditional towns). *Pueblos jóvenes* are low-income hillside squatter settlements founded over the past 60 years [17],[18]. *Pueblos tradicionales* tend to be in lower-lying flat areas, are inhabited by higher-income landowners, and date back to the late 19^th^ or early 20^th^ century. (See Figure 1 for photos of the two types of communities.) Because preliminary data from our research group indicated that *T. infestans* prevalence differed between these two types of towns [12], we compared migration and settlement patterns in 3 *pueblos jóvenes* and 2 *pueblos tradicionales*.

### Study participants

The research team worked with 2–3 community leader “gatekeepers” in each community to ensure acceptance and to recruit people who could provide detailed information about their personal history of migration and settlement (for interviews) or community history (for focus groups). A total of 94 female and male participants were enrolled in the study.

### Data collection activities

This was a qualitative study that employed focus group discussions and in-depth interviews. Focus group sessions were carried out with 8–10 participants in each community at central, well-known locations (health establishments, community centers) selected by the gatekeepers. We used participatory methodologies to explore the community's demographic characteristics and mobility patterns, and historical and current presence of triatomine vectors. Participants created community maps [19] which formed the basis for discussions of the history and characteristics of communities. Participants then created a timeline [19] of important community events dating back approximately 40 years, first exploring general events and then focusing on events related to Chagas disease and vector infestation. All sessions were audio-recorded; participatory activities were recorded on large sheets of paper that were hung on the wall and visible to all participants.

In-depth interviews utilized an event history calendar (EHC), a highly structured but flexible interview style that facilitates recall by using the individual's own experiences as cues [20]. Interviews were carried out with 10 participants per community, and explored migration history, experience with Chagas disease, the presence of its vectors, and customs of raising animals in each place of residence, starting from birth. Each interview, held at a location selected by the participant (their home, the focus group location), was audio-recorded and lasted 45 to 60 minutes. All interviews and focus groups were conducted by two of the authors (AB and GH).

Presence of vectors was examined by asking participants when they had seen triatomine bugs. Because *T. infestans* is the sole insect vector for Chagas disease in southern Peru, we only asked participants to recall the presence of this species. All of the study communities had been involved in insecticide application programs run by the Ministry of Health (MOH) and as a result were broadly familiar with *T. infestans*, which they refer to as a “chirimacha.” In this context, there is no other bug that goes by the same name. We also showed images of *T. infestans* to participants to aid their recall. During interviews, participants were asked if they recalled seeing triatomine bugs in their house or in their community for the specific years they lived in each place of residence. During focus groups, participants were asked to reach a consensus regarding the year that triatomine bugs first appeared and the areas most infested.

Data on the number of households, estimated population, and year of insecticide application were collected by our research group in collaboration with the Arequipa Regional Office of the Ministry of Health (MOH). The domiciliary infestation index (DII) is a community level variable equivalent to the number of infested houses divided by the total number of houses surveyed. We used ArcView 9.1 (ESRI) and existing maps to estimate the distance of each community from Arequipa and its surface area (to calculate population density).

### Data analysis

Detailed notes were taken from focus group audio-recordings and the large sheets of paper, and synthesized into matrices in Excel by theme and by community to carry out further analysis. Audio-recordings of interviews were transcribed verbatim from digital audio recorders to word processing programs and analyzed using the grounded-theory approach. Grounded theory refers to theory that is developed inductively from a body of data, in contrast to theory that is derived deductively from grand theory and not necessarily based on data. The grounded theory approach is applied by reading textual data, in this case transcripts and field notes, in order to discover the main concepts or themes that were mentioned during data collection, allowing the data to reveal its message (or theory) instead of looking for confirmation of a previously-developed hypothesis [21],[22],[23],[24]. Further information about grounded theory can be accessed via the web links listed in the references [25],[26]. Two authors (AB and GH) created a code set based on the main themes that emerged in the interviews after an initial reading of the transcripts. The Atlas-ti software (Scientific Software Development GmbH, 2005) was used to apply the codes to each interview transcript. The event history calendars themselves were also analyzed by entering the information into a single timeline in Excel, such that each decade from 1900 to the present year detailed the places of residence of participants and the presence of triatomine vectors in those places. The superimposition of the EHCs on a timeline allowed us to visualize patterns in migration and vector presence over time. Each coded interview was analyzed together with the interviewee's EHC and all focus group and interview data were analyzed by community and then across communities.

During the EHC interviews, all moves were recorded, including a change of abode within the same community. For the purposes of this analysis, only a change of resident community for one month or more, whether temporary or permanent, was considered as a movement.

## Results

**Figure 2:**
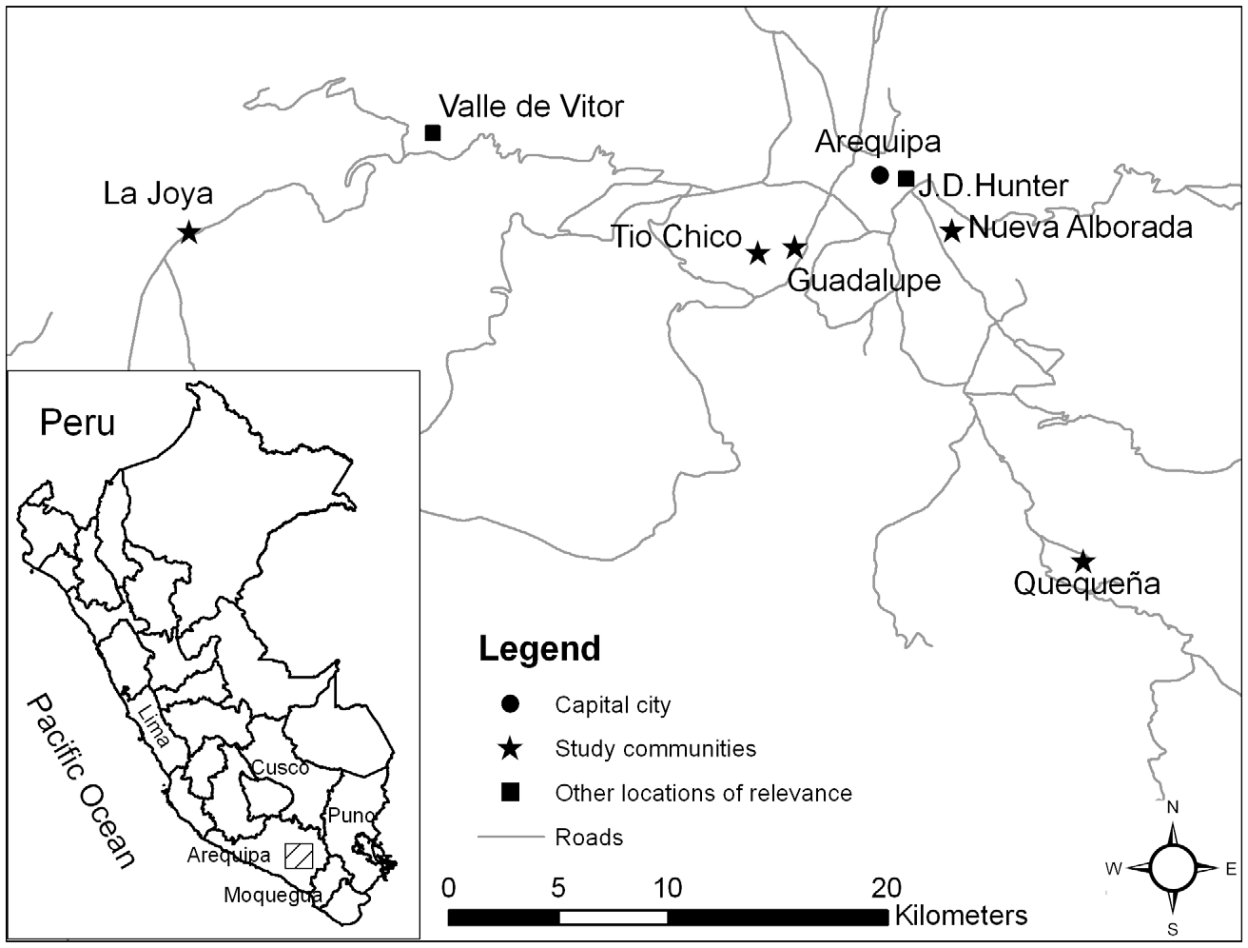
Map of study and non-study communities.

Key demographic and *T. infestans* infestation data from the 5 study communities are listed in Table 1, and their geographic location in reference to the urban center is shown in Figure 2.

**Table 1:** Demographic characteristics and vector prevalence in study communities.

Community	Number of households	Estimated population	Population density (inhab/km^2^)	Level of in-migration	DII (%)*	Households with *T. cruzi*-infected *T. infestans* (%)
***Pueblos jóvenes***						
Nueva Alborada	499	3,000	14,932.65	High	35.68%	0.00%
S. Maria de Guadalupe	315	1,600	13,060.90	High	44.59%	19.32%
Villa La Joya^1^	252	1,020	10,357.75	High	42.06%	14.68%
***Pueblos tradicionales***						
Quequeña	192	1,150	1,738.09	Moderate	19.19%	12.21%
Tío Chico	94	550	3,331.78	Low	0.00%	0.00%

*: DII = Domiciliary Infestation Index, number of infested houses divided by the total number of houses surveyed in the community.

1 Villa La Joya existed under other names prior to its official founding in 1978. In earlier years, however, it was much less urbanized and dense.

### Demographics and migration overview

Fifty people provided in-depth interviews and 44 participated in focus groups. Interview participants had a median age of 52 and 47 years for males and females, respectively, with a range of 20 to 80 years. Focus group participants had a median age of 67 for males and 49 for females, with a range of 20 to 79 years (Table 2). Older community members were purposely recruited for focus groups, to ensure knowledge of historical events.

**Table 2:** Demographic characteristics of participants of in-depth interviews (IDI) and focus groups (FG).

Community		Study activity	# Males	# Females	Median age in years [IQR]
***Pueblos jóvenes***	Nueva Alborada	IDI	2	8	38.0 [33.0–45.0]
		FG	1	9	43.5 [34.0–60.0]
	S. Maria de Guadalupe	IDI	4	6	46.0 [42.0–57.0]
		FG	0	6	45.0 [36.3–56.0]
	Villa La Joya	IDI	3	7	53.0 [41.0–56.0]
		FG	1	9	60.0 [49.0–63.0]
***Pueblos tradicionales***	Quequeña	IDI	4	6	48.0 [37.0–54.0]
		FG	4	5	39.0 [36.0–42.0]
	Tío Chico	IDI	2	8	50.0 [48.0–53.0]
		FG	0	10	53.5 [38.5–61.5]
**All communities**		IDI	15	35	47.0 [38.2–54.8]
		FG	6	39	49.0 [35.0–60.5]

Males moved more frequently than females, with a median of 3.0 (Interquartile range (IQR) 1.0–4.0) lifetime moves for females and 4.0 (IQR 2.5–5.5) for males. There was also more migration among residents of *pueblos jóvenes*: 80% of participants from *pueblos jóvenes* were in-migrants, while 60% of participants from *pueblos tradicionales* were non-migrants (Table 3). Typical patterns of migration are shown in Table 4. Residents of *pueblos jóvenes* typically moved from a rural birth community to the Arequipa area early in life due to economic stress. Later moves took them to a *pueblo jóven* near Arequipa in search of cheap housing and then multiple, short-term moves continued throughout life in search of work. Some residents of *pueblos tradicionales* also moved from a rural area to Arequipa during childhood or adolescence, usually for schooling. Later moves were few since these residents tended to settle in the *pueblo tradicional* to focus on building agricultural enterprise and raising a family. The majority of participants who migrated from rural areas recalled raising farm animals in their birth places, and many continued to raise animals, especially guinea pigs, rabbits and poultry, in peri-urban Arequipa.

**Table 3:** Migration experience of in-depth interview participants in *pueblos jóvenes* and *pueblos tradicionales*.

Interview participants' place of residence	# Migrants (%)	# Non-Migrants (%)	Total
*Pueblos jóvenes*	24 (80)	6 (20)	30
*Pueblos tradicionales*	8 (40)	12 (60)	20

**Table 4:** Example migration profiles for *pueblo joven* and *pueblo tradicional* residents.

Participant		Birth	Moves		
**Female, 37 years old, ** ***pueblo joven***	**Move # (Age)**	**Birth**	**Move 1 (Age 14)**	**Moves 2–6 (Ages 15–19)**	**Move 7 (Age 20)**
	**Location**	Moquegua	Arequipa (city center)	Arequipa (different peri-urban communities)	Nueva Alborada
	**Reason**		Father died at age 10, family fell into extreme poverty. Moved alone to seek work	Moved from house to house providing domestic work services	Became pregnant, married and moved with spouse and child
**Female, 53 years old, ** ***pueblo tradicional***	**Move # (Age)**	**Birth**	**Move 1 (Age 6)**	**Move 2 (Age 7)**	—
	**Location**	Lima (mining camp)	Tiabaya (peri-urban Arequipa)	Tío Chico	—
	**Reason**		Moved back to Arequipa with parents and siblings since father lost job	Moved with parents and sibling to buy land	—
**Male, 32 years old, ** ***pueblo joven***	**Move # (Age)**	**Birth**	**Moves 1–3 (Ages 12–17)**	**Move 4 (Age 18)**	**Move 5 (Age 20)**
	**Location**	Arequipa (peri-urban community)	Arequipa (different peri-urban communities)	Moquegua (southern Peru)	Nueva Alborada
	**Reason**		Moved from house to house, of relatives and of others	Voluntary military service	Moved in with mother
**Male, 65 years old, ** ***pueblo tradicional***	**Move # (Age)**	**Birth**	**Move 1 (Age 18)**	**Move 2 (Age 58)**	—
	**Location**	Bolivar (city center of Arequipa)	San Gerónimo (city center of Arequipa)	Tío Chico	—
	**Reason**		Moved due to earthquake, to area with anti-seismic constructions	Moved to live in a quiet, less polluted place	—

### Migration histories in *pueblos jóvenes*

Study participants indicated that the *pueblos jóvenes* of Arequipa's urban periphery were mostly settled by people from greater Arequipa and the northern Andean regions of Cusco and Puno, and in lesser numbers by people from the southern coastal/Andean region of Moquegua (see map insert in Figure 2). The formal founding dates of the *pueblos jóvenes* in this study ranged from 1970 to 1981, although some residents had lived in these settlements since the 1960s. Migration from rural birthplaces to urban Arequipa was motivated by acute economic stress, with a few families sending their children away to work as early as age six. This early move was followed by migration later in life to a *pueblo joven* to acquire land and housing. Each quote presented in the Results section is followed by the participant's sex, age, type of current community, and the years corresponding to the movement. Two examples of interviewees who moved alone as children follow:

My father died when I was ten years old… All of his children went [from Moquegua] to different places and we didn't live together anymore… [At age 14]… I went to Arequipa… because we didn't have support anymore… My mother was all alone so I decided to come and work. (Female, 39 years old, *pueblo joven*, 1969–1973)

I came [to Arequipa] because of poverty [when I was eight years old]… because of poverty. My mother, my father, suffered [in Puno]… At age 15 I saw my father, my mother again. (Male, 65 years old, *pueblo joven*, 1951)

Other participants moved with their entire family for work:

I lived [in Puno] until I was at least three years old. I came from there in order to live in Arequipa… My mother, my father brought me here… They told me that… before there was life there [in Puno]… but sometimes it rains and sometimes it doesn't rain and there is no work. (Male, 41 years old, *pueblo joven*, 1967)

Migration to *pueblos jóvenes* enabled early settlers to acquire land at little cost as squatters invading land. The *pueblo tradicional* of Tío Chico is located just below several hillside *pueblos jóvenes*, including Guadalupe:

My father came to work with another boss… who lived in Tío Chico… His old boss told us to leave his house… We had to look for another place since we were renters… At that time these hills (the *pueblo joven* of Guadalupe, located above Tio Chico)… didn't have any inhabitants… so they created an invasion… My father did it with several people… mainly people from Tío Chico… so he got a lot (land parcel) where we could live. (Female, 47 years old, *pueblo joven*, 1970)

By contrast, residents who arrived after the original invasion bought an existing house or land parcel, rented a house or room, or lived with family members. *Pueblos jóvenes* are still expanding in geographic area and population, and currently are composed of multiple generations, including the original migrants, their children and grandchildren, and newly-arrived migrants.

In blocks 1, 2, 3 and 4, people come from Juliaca [in department of Puno], Puno, Cusco, mainly from the Sierra (Andes region)… In blocks 5 and 6 live the children of the people who came [to Guadalupe] first or the children of the older people who live in nearby communities. (Focus group participants, *pueblo joven*, 2008)

### Migration histories in *pueblos tradicionales*

*Pueblos tradicionales* are made up primarily of people who have lived in the community since birth and whose families have lived there for several generations. As in *pueblos jóvenes*, the founding migrants arrived in search of land and housing. However they usually purchased this higher-cost land:

We moved because my father lost his job as a miner… They fired him and he was from Arequipa… My father… bought land here in Tío Chico. (Female, 53 years old, *pueblo tradicional*, 1962)

Later migrants tended to move to *pueblos tradicionales* motivated by a return to family roots or the search for a calmer environment:

I lived in the center [of Arequipa] until… seven years ago when I came here, because there was too much pollution, smoke, noise… We decided to come here… to the house my father built. (Male, 66 years old, *pueblo tradicional*, 2001)

Current migration dynamics are causing important changes to the population of *pueblos tradicionales*. Many people, especially young people, are moving out of *pueblos tradicionales* due to a lack of opportunities. In the two *pueblos tradicionales* in this study, out-migration has resulted in diminished populations and a shortage of agricultural workers. To compensate, land owners hire temporary workers who are often migrants to the peri-urban areas of Arequipa. In Tío Chico, these workers do not live in the town itself since there are few available houses and they can live more affordably in the surrounding *pueblos jóvenes*. In contrast, Quequeña, being further from the city, lacks housing options other than the town itself. Rental is therefore common since the property owners have moved and need people to watch over their properties and work their land. As a result, Quequeña is experiencing relatively recent in-migration, while Tío Chico is not.

### Migration histories common to both *pueblos jóvenes* and *pueblos tradicionales*

A constant across most participants' adult lives was migration to live with a partner or spouse and children:

First my husband brought me to Tío Chico. We were there for a month before we went to Ayacucho (south-central Andes)… where we spent one year before we went to Lima… because my husband worked in construction and they sent him from one place to another… Then we came back to Arequipa, [to Tío Chico]. (Female, 50 years old, *pueblo tradicional*, 1975–1977)

My boss died of a heart attack… She was like my mother… so I got married quickly… We went to live on another farm… Then we moved again to this town and lived as renters… But then it was too much for the rent since we only worked sometimes… so we preferred to get a lot (land parcel) since we already had children… and otherwise we didn't have enough to live. (Female, 57 years old, *pueblo joven*, 1966–1974)

Across communities, the search for work was a constant that often spanned generations, with migration in early life by parents and in later life by participants. These participants described the almost constant movement of their fathers in search of work:

[My father] used to cut trees and then he'd go out… with … companies that hire personnel… He was a carpenter… They'd hire him for six months, seven months, eight months and then the work contract would finish and he'd come back… He went to Tacna (southern coast), to mines in Catanga (in Cusco) [and mines in] Quellaveco, to the north (in Moquegua). (Female, 53 years old, *pueblo tradicional*, 1962–1982)

Male participants described their own search for work opportunities and female participants discussed similar searches by their partners:

I went to the army… in Arequipa… I still didn't have a home. I lived here and there… I dedicated myself to working in mines… I've been in Chuquibamba (Arequipa region)… in Ayacucho (south-central Andes)… in Huánuco (central Peru, jungle/Andes juncture)… I worked sporadically… [In between] I rested… I would come here [to Quequeña] to visit my family, see friends, get land for my house. (Male, 63 years old, *pueblo tradicional*, 1976–1990)

[My husband] would come home every two to three months… [He'd go] to Mollendo (Arequipa region)… like every young man that wants to make his life… Now he goes once or twice a year… He used to go to Pucallpa [in the jungle] to the north… Now he goes for thirty days once or twice a year. (Female, 53 years old, *pueblo jóven*, 1981–2008)

### The presence of triatomine vectors according to participant memories and MOH data

**Figure 3:**
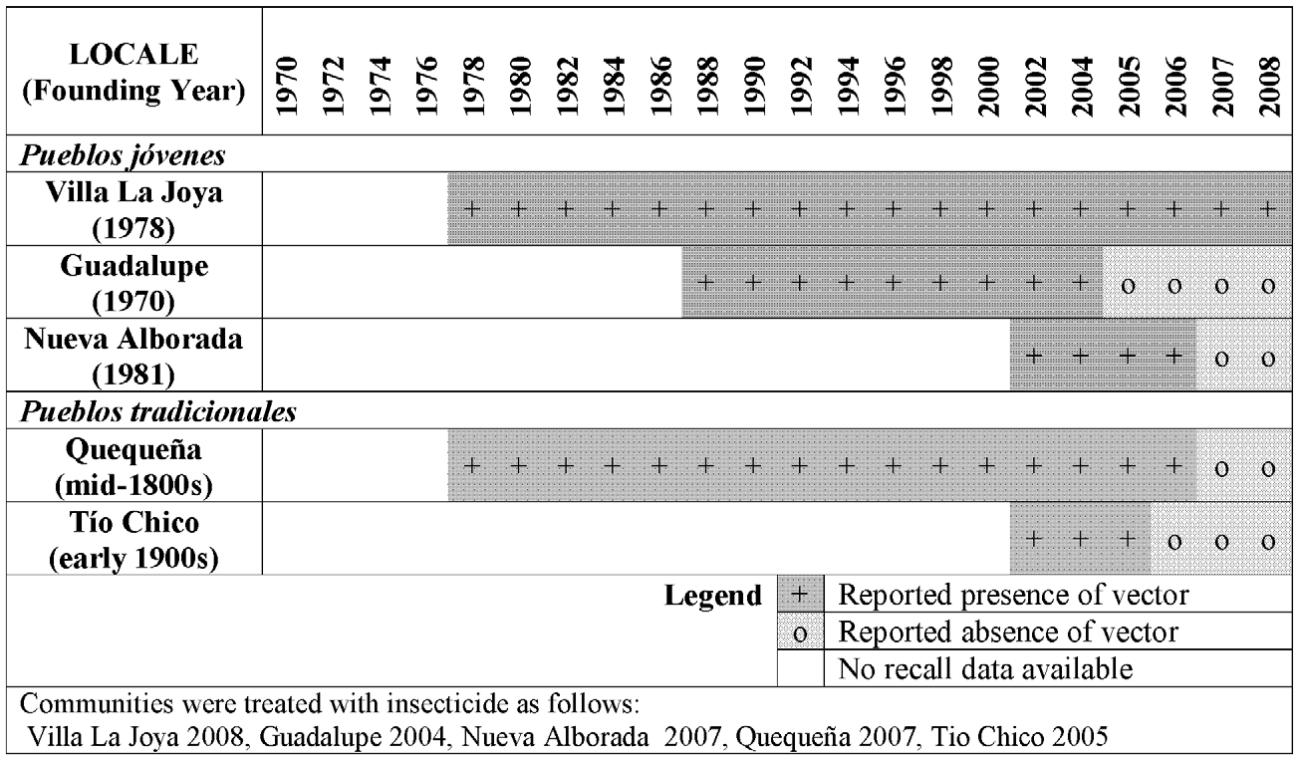
Timeline of reported presence (+) or absence (o) of vectors in study communities.

Forty-six (92%) of 50 interviewees had seen triatomine vectors (locally known as *chirimachas*) during their lifetimes in some place of residence; the four who did not report seeing *chirimachas* lived in the *pueblo tradicional* of Tío Chico (Figure 3). Participants reported seeing no triatomines in the highland Andes regions of Cusco and Puno, where many migrants were born. In addition, the urban center of Arequipa (often the first place of residence for low-income migrants to Arequipa) was described as vector-free.

The earliest sighting of triatomines occurred in Moquegua in 1942, a sending area for some migrants to urban Arequipa that is located south of the study communities (Figure 2). Other early memories came from the rural areas of Valle de Vitor and La Joya, both valleys west of Arequipa. The study community of Villa La Joya is a *pueblo joven* founded near the more rural and longer established community of La Joya. Villa La Joya had its first residents in the early 1960s, but *chirimachas* were not memorable until the town was much more populated in the late 1970s and 1980s.

Closer to the urban center, reported vector presence showed a similar pattern of infestation following settlement, but much more recently. The first memories of urban vector presence from study participants were from the *pueblo joven* of Jacobo D. Hunter in 1968, a peri-urban district settled in early squatter invasions. Years later, in 2002, Hunter was the setting of a highly publicized child death due to acute Chagas disease [27]. Peri-urban *pueblos jóvenes* increased in number and size during the 1960s and 1970s, and vector presence was reported in the urban *pueblos jóvenes* of our study roughly 20 years following original settlement. Participants from Guadalupe (founded in 1970) and Nueva Alborada (founded in 1981) noted the first widespread appearance of triatomines in 1988 and 2002, respectively.

In *pueblos tradicionales*, vector reports followed a different pattern than in the *pueblos jóvenes*. Although their settlement dates back to the mid-1800s, focus group participants recalled first seeing vectors in Quequeña around 1978. They noted that *chirimachas* were especially concentrated in the area of town around a communal stable and zones of relatively newer settlement by migrants. Tío Chico, in contrast, had very few reports of infestation, and much later (from 2000). The following quotes contrast the degree of infestation between Tío Chico and Guadalupe, the hillside *pueblo joven* located above Tío Chico. Participants specifically associated infestation with the presence of domestic animals.

P1: I haven't seen any *chirimachas*… Even when they fumigated, I didn't see any.

P2: In my house, in one of the walls outside where there are a lot of animals, there were a lot of *chirimachas*… but my house was the only one…

P3–9: I've never seen them either.

(Focus group participants, Tío Chico, 2000–2006)

P1: There were *chirimachas* in all of Guadalupe.

P2–6: Yes!! A lot! Everywhere!

P2: For example, I didn't have animals in my house, but my neighbor did, and since my wall wasn't stuccoed, the *chirimachas* would come in.

(Focus group participants, Guadalupe, 1990–2004)

Residents reported continuous presence of *chirimachas* in all study communities except Tío Chico until insecticide spraying occurred (Figure 3).

According to MOH vector control data, the *pueblos tradicionales* in our study had considerably lower infestation rates than *pueblos jóvenes* (Table 1). The high infestation rates in *pueblos jóvenes* parallel higher population densities of 10,300–15,000 inhabitants/km^2^, compared to 1,700–3,300 inhabitants/km^2^ in *pueblos tradicionales* (Table 1).

## Discussion

The prevalence of Chagas disease varies widely among communities around Arequipa. High *T. cruzi* infection rates in children, reflecting recent transmission, were documented in several recently-formed *pueblos jóvenes*, while long-established *pueblos tradicionales* have almost no infection in children [12]. The proximate cause of infection heterogeneity is the difference in triatomine infestation rates [12],[28]. We show here how migration and associated activities contribute to conditions promoting infestation in *pueblos jóvenes*. Human migration patterns thus constitute an important underlying determinant of Chagas disease risk in and around Arequipa.

Most migrants to *pueblos jóvenes* came from areas without infestation and are unlikely to have brought the vector with them. However, community members later made multiple short- to medium-term moves, often to the valleys west of Arequipa for seasonal agricultural labor. These valleys are also the best-known historical foci of *T. cruzi* transmission in the region [29]. Migrants may have become infected while living temporarily in the valleys, or may have carried vectors back to their long-term communities in their belongings.

An alternative, not mutually exclusive hypothesis is that vectors were always present in *pueblos tradicionales* prior to the construction of *pueblos jóvenes*, but not in large enough numbers to be a memorable event for study participants. When *pueblos jóvenes* quickly took over peri-urban hillsides, existing vector populations may have exploded. Rural migrants from highland areas brought their domestic animal husbandry practices to the city with them, raising small animals such as guinea pigs and rabbits for sale or for personal consumption, and keeping them in small yards close to human dwellings. At the time of vector control spraying in 2004, the *pueblo joven* of Guadalupe contained a total of 5,006 domestic animals (predominantly guinea pigs, rabbits, poultry and dogs, but also sheep and cows); the presence of guinea pigs, in particular, was associated with an increased risk of infestation both in the animal enclosure and in the adjacent human house [13]. The high density of animals provides an abundance of blood meal sources to support large vector populations, and potentially contributes to Chagas disease transmission, since all except poultry are susceptible to *T. cruzi* infection.

Interestingly, one *pueblo joven*, Nueva Alborada, was highly infested with vectors, but none of the 1,460 insects examined during the Ministry insecticide application campaign were carrying *T. cruzi* [30]. Focus group participants in Nueva Alborada reported *chirimachas* in the community only back to 2000, while focus groups in the other two *pueblos jóvenes* recalled insects in their communities for a much longer period. It is possible that, given the short history of vector infestation in Nueva Alborada, the parasite has yet to be successfully introduced into this community. In contrast, in Guadalupe and Villa La Joya, the longer history of infestation may have led to single or multiple introductions of the parasite in these communities. The relationship between time since introduction of the vector and presence of *T.cruzi* merits further epidemiological investigation.

Two final considerations include the possible transformation of long-standing *pueblos tradicionales* by an influx of low-income migrants and the urbanization of rural areas. Although many of the original residents had emigrated from both traditional towns, we observed increased population density and levels of in-migration that were similar to those in *pueblos jóvenes* only in Quequeña, where low-income migrants are filling the population void. In contrast, no new migrants had moved to Tío Chico, because low-income farm workers who cultivate fields surrounding Tío Chico can live more cheaply in a nearby *pueblo joven*. The case of Quequeña draws attention to a previously undescribed phenomenon of population loss and replacement by low-income migrants, creating the human settlement pattern of a *pueblo jóven* within a *pueblo tradicional*, and providing insight into why *T. infestans* and *T. cruzi* are prevalent in Quequeña.

These complex patterns of human migration and triatomine infestation demonstrate the conditions underlying the urbanization of Chagas disease in Arequipa. Factors common to the infested *pueblos jóvenes* include recent, rapid settlement, high human and animal density, and frequent temporary migration to Chagas disease-endemic areas. However, some *pueblos tradicionales* are also undergoing similar processes. As peri-urban areas in South America continue to grow, vector control programs must remain vigilant against reinfestation, as well as infestation of communities not previously recognized to be at risk, such as the *pueblos tradicionales* of Arequipa. In addition, our data point to at least three potential interventions for improving vector control in Arequipa: 1) Intensifying vector surveillance efforts in areas with highly mobile populations; 2) Creating educational campaigns for migrant workers to Chagas-endemic areas; and 3) Fomenting collaboration between the Arequipa Region's Ministry of Health and Ministry of Housing to monitor the emergence of new *pueblos jóvenes* for their inclusion in the vector surveillance system.

## Supporting Information

Alternative Language Abstract S1 Translation of the abstract into Spanish by AMB and GCH.
(0.03 MB DOC)
