# Supplementary material for: Chagas Disease, Migration and Community Settlement Patterns in Arequipa, Peru
Source: PLoS Negl Trop Dis. 2009 Dec 15;3(12):e567. doi: 10.1371/journal.pntd.0000567 (PMC2790340; doi:10.1371/journal.pntd.0000567)
Supplement: Alternative Language Abstract S1 — Translation of the abstract into Spanish by AMB and GCH. (0.03 MB DOC) [file pntd.0000567.s001.doc]

**Chagas Disease, Migration and Community Settlement Patterns in Arequipa, Peru**

Bayer AM, Hunter G, et al

**Resumen**

**Antecedentes**

La enfermedad de Chagas es una de las enfermedades tropicales olvidadas más importantes en las Américas. La transmisión de Chagas por vectores ha sido históricamente poco frecuente en contextos urbanos. Sin embargo, en comunidades marginales cerca a la ciudad de Arequipa, Perú, se han establecido ciclos de transmisión urbanos. En este estudio, examinamos la historia de los patrones de migración y asentamiento en estas comunidades, y sus conexiones con la transmisión de la enfermedad de Chagas.

**Metodología/Resultados Principales**

Este fue un estudio cualitativo que utilizó discusiones en grupo y entrevistas a profundidad. Se llevaron a cabo cinco discusiones en grupo y cincuenta entrevistas a profundidad con 94 miembros comunitarios de 3 pueblos jóvenes y 2 pueblos tradicionales cerca a Arequipa, Perú. Las discusiones en grupo utilizaron metodologías participativas para explorar los patrones de movilidad y la presencia histórica y actual de vectores triatominos en la comunidad. Las entrevistas a profundidad se basaron en calendarios de historia de acontecimientos (“event history calendars”) para explorar con los participantes su historia de migración y experiencias con la enfermedad de Chagas y sus vectores. Se analizaron los datos de las discusiones en grupo utilizando metodologías específicas para información participativa y se codificaron y analizaron los datos de las entrevistas utilizando la metodología de teoría fundamentada (“grounded theory”). Los datos entomológicos fueron recolectados durante la campaña actual de control de vectores. Encontramos que es poco probable que los migrantes a los pueblos jóvenes en Arequipa trajeron los triatominos a la ciudad al momento de su llegada. Sin embargo, mudanzas estacionales temporales llevaron a los residentes de pueblos jóvenes a los valles alrededor de Arequipa donde los vectores son prevalentes. Adicionalmente, el patrón de asentamiento de los pueblos jóvenes y la práctica de los residentes de criar animales domésticos crea un ambiente favorable para la proliferación y dispersión de los vectores. Finalmente, descubrimos dentro de un pueblo tradicional un fenómeno de pérdida y reemplazo de la población por migrantes de bajos ingresos, el cual ha creado el patrón de asentamiento humano de un pueblo joven nuevo dentro de esta comunidad tradicional.

**Conclusiones/Significancia**

El patrón de migración humana es un determinante subyacente importante en el riesgo de la enfermedad de Chagas dentro y cerca de la ciudad de Arequipa. La migración estacional frecuente por parte de los residentes de pueblos jóvenes peri-urbanos crea un sendero de introducción de vectores a estas comunidades. Las dinámicas demográficas cambiantes de pueblos tradicionales también están contribuyendo a condiciones favorables para la transmisión de la enfermedad de Chagas. Los programas de control necesitan incluir vigilancia de infestación en comunidades anteriormente asumidas de estar libres de vectores.
